# Adductor canal block versus femoral nerve block for total knee arthroplasty: a meta-analysis of randomized controlled trials

**DOI:** 10.1038/srep40721

**Published:** 2017-01-12

**Authors:** Duan Wang, Yang Yang, Qi Li, Shen-Li Tang, Wei-Nan Zeng, Jin Xu, Tian-Hang Xie, Fu-Xing Pei, Liu Yang, Ling-Li Li, Zong-Ke Zhou

**Affiliations:** 1 Department of Orthopedics, West China Hospital/West China School of Medicine, Sichuan University, Chengdu, 610041, P.R. China; 2 Department of Prosthodontics, West China College of Stomatology, Sichuan University, Chengdu 610041, China; 3 Department of Breast Surgery, West China Hospital/West China School of Medicine, Sichuan University, Chengdu, 610041, P.R. China; 4 Center for Joint Surgery, Southwest Hospital, Third Military Medical University, Chongqing 400038, P.R. China; 5 Tianjin hospital, Tianjin, 300041, P.R. China

## Abstract

Femoral nerve blocks (FNB) can provide effective pain relief but result in quadriceps weakness with increased risk of falls following total knee arthroplasty (TKA). Adductor canal block (ACB) is a relatively new alternative providing pure sensory blockade with minimal effect on quadriceps strength. The meta-analysis was designed to evaluate whether ACB exhibited better outcomes with respect to quadriceps strength, pain control, ambulation ability, and complications. PubMed, Embase, Web of Science, Wan Fang, China National Knowledge Internet (CNKI) and the Cochrane Database were searched for RCTs comparing ACB with FNB after TKAs. Of 309 citations identified by our search strategy, 12 RCTs met the inclusion criteria. Compared to FNB, quadriceps maximum voluntary isometric contraction (MVIC) was significantly higher for ACB, which was consistent with the results regarding quadriceps strength assessed with manual muscle strength scale. Moreover, ACB had significantly higher risk of falling versus FNB. At any follow-up time, ACB was not inferior to FNB regarding pain control or opioid consumption, and showed better range of motion in comparison with FNB. ACB is superior to the FNB regarding sparing of quadriceps strength and faster knee function recovery. It provides pain relief and opioid consumption comparable to FNB and is associated with decreased risk of falls.

Total knee arthroplasty (TKA) is regarded as an effective treatment for end-stage knee osteoarthritis^1^,^2^. More than 670,000 TKAs were performed annually within the United States alone^3^. With the aging population, the number of TKAs is expected to increase, which highlights a relevantly heavier burden on healthcare. Over 50% of post-TKA patients suffer from moderate-to-severe postoperative pain, which results in immobility-related complications and prolonged hospitalization^4^,^5^. Therefore, effective analgesia is of paramount importance for post-TKA patients. Femoral nerve block (FNB) is known to provide superior pain control and shortens the time of functional recovery and the length of hospital stay without associated side effects, in comparison with epidural or intravenous patient-controlled analgesia (PCA)^6^,^7^. However, findings from previous studies show that FNB reduces quadriceps muscle strength and results in an increased risk of falls^5^,^8^,^9^. The introduction of fast-track clinical pathways leads orthopedic surgeons to question risk–benefit ratio for FNB and explore an alternatively optimal analgesia modality, providing motor strength preservation with effective analgesia, to enable faster rehabilitation, shorter hospitalization, and earlier ambulation^8^,^9^,^10^,^11^.

Adductor canal block (ACB) is a relatively new alternative for post-TKA pain management. Regional anesthesia is deposited within an adductor canal that can be easily visualized at the middle third of the thigh with use of ultrasonography. Consequently, ACB can be performed with a high success rate. Anatomical study of adductor canal showed that an adductor canal contained multiple afferent sensory nerves (e.g. saphenous nerve, medial femoral cutaneous, and medial retinacular nerve etc.) but only a single efferent motor nerve (vastus medialis of the quadriceps muscle) that potentially affected motor function^12^,^13^,^14^. Therefore, ACB may have a minimal effect on quadriceps muscle strength, but provides a comparable level of pain relief and early mobilization.

Several randomized controlled trials (RCT) have compared ACB to FNB. Many of these trials contained relatively small cohorts, and demonstrated inconsistent outcomes^15^,^16^,^17^,^18^,^19^,^20^. This uncertainty leads to the determination of which peripheral nerve blockade to adopt by the preference of the surgeons. Two meta-analyses were published on this topic recently^21^,^22^. Dong *et al*. reported that ACB shows no superiority than FNB group regarding muscle strength and pain control^21^. However, Li *et al*. demonstrated that ACB preserved greater quadriceps strength more than FNB with similar pain control^22^. Nevertheless, these two meta-analyses included several non-RCTs, and thereby their results should be treated with caution.

Several more RCTs on this subject have been published without conclusive results^20^,^23^,^24^,^25^. In addition, Li *et al*. (2015) conducted a recent meta-analysis of RCTs showing that ACB achieves better analgesic effects comparing with FNB^26^. However, it contained some methodological shortcomings, errors in inclusion criteria and data extraction, and high heterogeneity. Not only did these studies have these limitations, but also their conclusions were inconsistent (Table 1). Considering all these issues, it is impossible to give clear advice on which method to adopt.

Thus, we undertook a further meta-analysis to evaluate whether ACB is superior to FNB with respect to: (1) muscle strength; (2) pain score at rest or mobilization; (3) clinical outcomes; and (4) complications. We hypothesized that ACB results in less quadriceps motor impairment and incidence of falling, but provide similar analgesia and opioid intake.

## Materials and Methods

The systematic review and meta-analysis was in accordance with the PRISMA (Preferred Reporting Items for Systematic Reviews and Meta-Analyses) guidelines^27^. Ethical approval was unnecessary in this study because it was a meta-analysis analyzing existing articles and did not need to handle individual patient data.

### Literature search

We utilized a predetermined protocol in meta-analysis (http://www.prisma-statement.org). We systematically searched PubMed, Embase, Web of Science, Wan Fang, China National Knowledge Internet (CNKI), and the Cochrane Database from inception to April 10, 2016, with the search terms: “adductor canal block” OR “saphenous nerve block” OR “ACB” AND “femoral nerve block” OR “FNB”AND “total knee arthroplasty” OR “total knee replacement” OR “TKA” OR “TKR”. We supplemented our search with: (1) bibliographies from the earlier systematic reviews; (2) ongoing prospective RCTs from the ClinicalTrials.gov website; (3) references mining of eligible publications. There were no language or publication data restrictions on trial eligibility.

### Inclusion criteria

To qualify for inclusion, the studies had to be randomized controlled trials comparing ACB with FNB in primary post-TKA patients. Any non-RCTs, quasi-RCTs, retrospective studies, cadaver studies, comments, letters, editorials, protocols, guidelines, surgical registries and review papers were excluded. Disagreements were resolved by consensus.

### Study selection

Study identification was conducted by the predefined eligibility criteria. After eliminating duplications, two reviewers independently screened the titles and abstracts of all studies identified by the search strategy and discarded those that were obviously ineligible. If suitability could not be determined, the full article was assessed. Discrepancies were reconciled through discussion.

### Data extraction

A predefined data collection form was developed to extract data from the eligible studies by two independent reviewers. Items collected were authors, publication date, patient demographics, the sample size of the patients, treatment regimens, dosages and types of anesthesia drug administered, the method of anesthesia and all clinical outcomes reported in eligible studies. The primary outcome measures were quadriceps strength and pain control, including maximum voluntary isometric contraction (MVIC) of quadriceps and adductor, muscle strength, and visual analog scale (VAS) pain scores at rest or mobilization. The improvement in clinical knee function and complications was regarded as secondary outcomes. The secondary outcomes were complications (risk of falls, vomiting and nausea etc.), mobilization ability (range of motion [ROM], Timed-Up-and-Go [TUG] test, the time to straight leg raising [SLR]) and opioid rescue consumption.

Corresponding authors of included studies were contacted via e-mail for relevant information if the available data were insufficient. Data in other forms (e.g. median, confidence intervals, or range of values) were converted to mean and standard deviation based on Cochrane Handbook. Discrepancies were resolved by consensus. When no consensus could be reached, a third reviewer cast the decisive vote.

### Assessment of methodological quality

Two authors assessed the quality of included studies independently with use of Cochrane Collaboration tool (domain-based risk-of-bias tables)^28^. We selected 6 domains related to risk of bias: random sequence generation, allocation concealment, blinding of participants and personnel, blinding of assessors, incomplete data, selective reporting and other bias^29^. The overall quality of each study was evaluated as “low risk of bias”, “high risk of bias”, or “unclear risk of bias”.

The quality of evidence for each finding was rated based on criteria established by the GRADE (Grading of Recommendations Assessment, Development and Evaluation) group^30^. The RCTs was considered as high-quality evidence, which could be downgraded to moderate, low, or very low quality for five reasons (high risk of bias, inconsistent results, indirect evidence, imprecision and publication bias). Any disagreement was settled by discussion among the research team.

### Statistical analysis

Review Manager 5.3 software was used for statistical analysis and a P-value < 0.05 was considered statistically significant. The odds ratios (OR) with 95% confidence intervals (CI) for ACB compared with FNB were calculated for dichotomous variables. If outcomes were measured in the same way between studies, we calculated mean differences (MD) and 95% CI for continuous variables. Heterogeneity was tested with use of the chi-squared test and I^2^ statistic. If significant (*P* < 0.1 or I^2^ > 50%), a random-effect model (REM) was used to estimate the overall effect sizes and a sensitivity analysis was carried out to investigate the potential sources of heterogeneity. Otherwise, fixed-effect model (FEM) was adopted. The Z test was used to evaluate the overall effect. In addition, publication bias was assessed by funnel plots.

## Results

### Study characteristics

**Figure 1:**
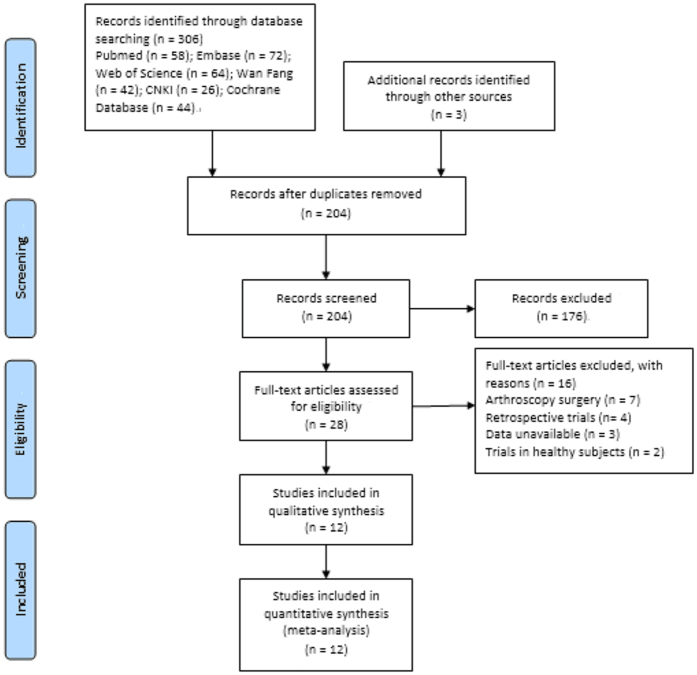
Flow chart showing study identification, inclusion and exclusion.

The process of study selection was showed in Fig. 1. Our search strategy identified 306 publications. Another 3 studies were identified through manual search. After removing duplications, scanning titles and abstracts, and reading the full-text, 12 RCTs met the inclusion criteria^15^,^16^,^17^,^18^,^19^,^20^,^23^,^24^,^25^,^31^,^32^,^33^.

Table 2 displayed the detailed characteristics of the studies. A total of 647 evaluable patients (647 knees) were available for analysis. The 12 eligible studies involved in 320 knees that underwent ACB and 327 knees that underwent FNB. All papers were published from 2013 to 2016. Inter-reviewer agreement for the data extraction and evaluation of the risk of bias did not reveal significance (kappa = 0.89).

### Risk of bias and quality of evidence

**Figure 2:**
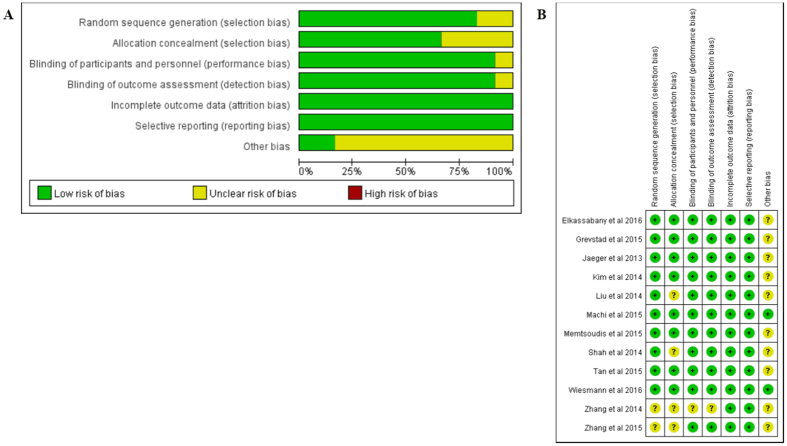
(**A**) Risk of bias graph; (**B**) for Risk of bias summary (“+” indicates a low risk of bias, “−” indicates a high risk of bias, “?” indicates unclear or unknown risk of bias).

The risk-of-bias assessments were displayed below and in Fig. 2. The sequence generation (randomization scheme used) was described fairly well in 10 studies, and allocation concealment in 8 studies; in the remainder, this information was absent or unclear^15^,^18^,^24^,^33^. 11 studies illustrated the blinding of assessor and participants explicitly, and the blinding was not described in one of studies^15^. The dropout or withdraw patients rate was lower than 20% in all studies. In addition, all trials reported the outcomes planned previously, and no trial received commercial funding to support their research. We did not find any other apparent bias in each included study. After examination, 8 of the included studies were assessed as having a low risk of bias, 3 of them had an unclear risk of bias, and one was evaluated as having a high risk of bias.

These findings were considered as being of moderate to high quality through GRADE approach. The presence of studies with unclear risk of bias and one study with high risk of bias downgraded the quality of evidence (data not shown)^15^,^18^,^24^,^33^.

### Primary outcomes

#### Muscle strength

**Figure 3:**
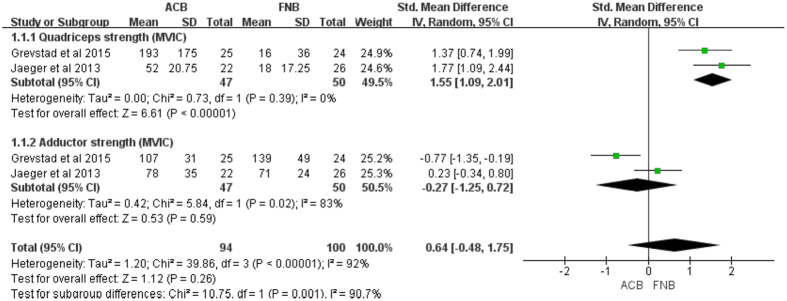
Forest plot of quadriceps and adductor MVIC between ACB and FNB. (MVIC, maximum voluntary isometric contraction).

Muscle strength was assessed as quadriceps and adductors MVIC, which was expressed as a percentage of baseline values after block at different follow-up time. There was moderate quality of evidence from two studies^16^,^17^ (97 knees) that no significant change in adductors MVIC was identified between ACB and FNB group (MD = −0.27, 95% CI: −1.25–0.72; *P* = 0.059) with moderate heterogeneity (I^2^ = 83%, *P* = 0.02, Fig. 3, Table 3).

In addition, there was high quality of evidence from two studies (97 knees) that ACB was superior to the FNB with regard to the change in quadriceps strength (MD = 1.55, 95% CI: 1.09–2.01; *P* < 0.0001) with no heterogeneity (I^2^ = 0%, *P* = 0.39, Fig. 3, Table 3). Quadriceps strength was also evaluated with use of manual 5-grade muscle strength scale. Considering that the origin of heterogeneity may be attributed to the duration of follow-up, subgroup analysis was conducted based on different follow-up time. The results showed that ACB was associated with greater quadriceps strength compared with FNB throughout most time measurements (e.g. postoperative, 4–6, 12, 24, 48, and 72 h) (Table 3). However, strength was not statistically significantly different between groups at 2 h^18^,^25^ (MD = 0.34, 95% CI: −0.23–0.90; *P* = 0.24) and 6–8 h^18^,^19^ (MD = 0.36, 95% CI: −0.04–0.77; *P* = 0.08) with moderate heterogeneity (Table 3).

#### Pain at rest or activity

**Figure 4:**
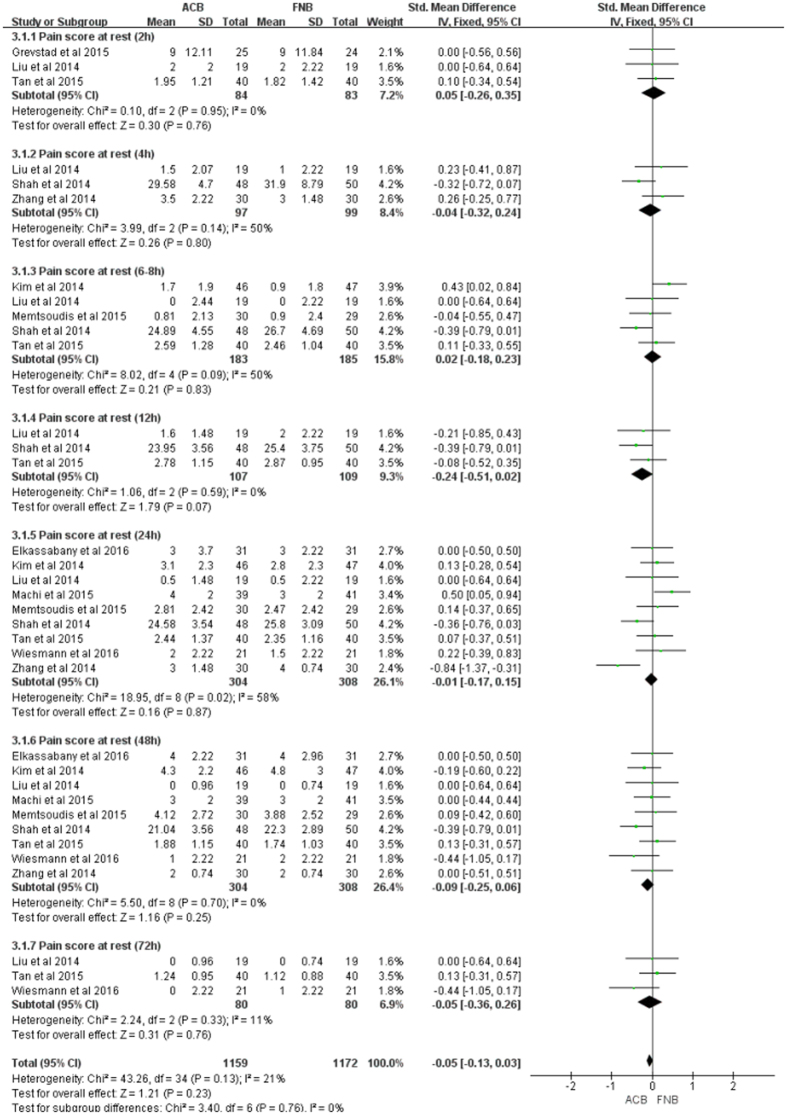
Forest plot of pain score at rest between ACB and FNB.

Data on 661 primary TKAs (including 329 with ACB and 322 with FNB) were pooled from 10 trials^15^,^16^,^18^,^19^,^20^,^23^,^25^,^31^,^32^,^33^ analyzing the pain score at rest (defined as VAS pain scores at different time points at rest). The ACB and FNB groups were not statistically significantly different with regard to pain during rest at each time point (e.g. post-anesthesia, 2, 4, 6–8, 12, 24, 48, and 72 h), and low to moderate heterogeneity was detected in this study (Fig. 4, Table 3).

**Figure 5:**
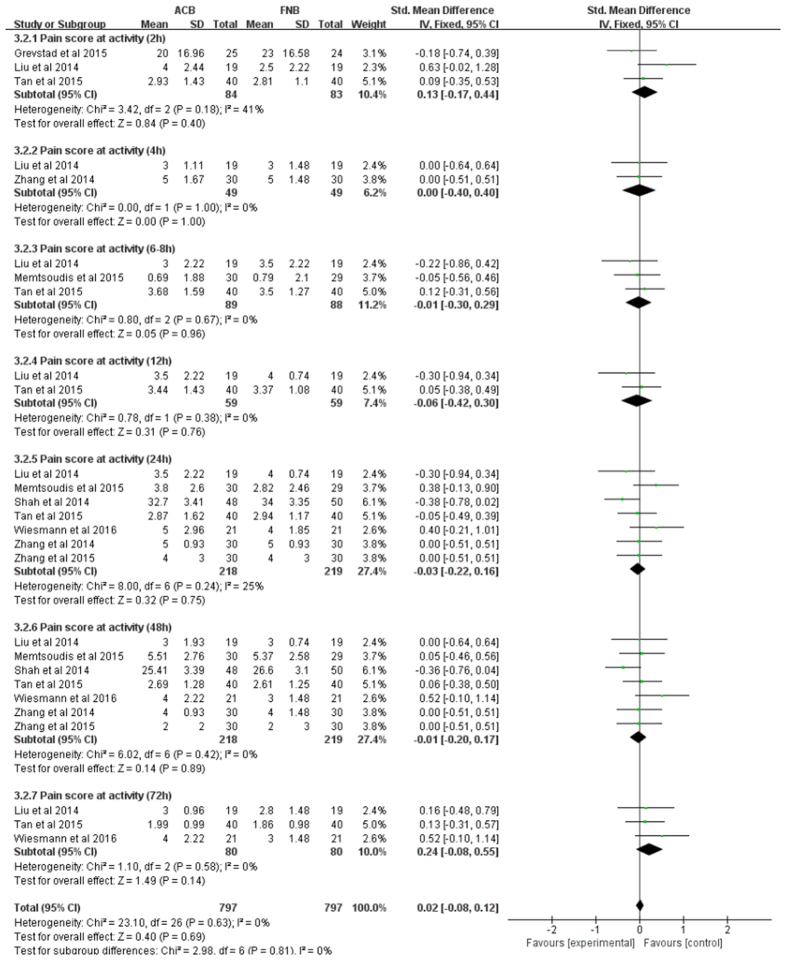
Forest plot of pain score at activity between ACB and FNB.

Data on 486 primary TKAs (including 243 with ACB and 243 with FNB) from 8 trials^15^,^16^,^18^,^19^,^20^,^24^,^25^,^33^ were analyzed with respect to pain score at activity. There were no statistically significant differences in pain at activity, between groups, at any time point (e.g. post-anesthesia, 2, 4, 6–8, 12, 24, 48, and 72 h) with low heterogeneity (Fig. 5, Table 3).

### Secondary outcomes

#### Mobilization ability

##### TUG test

TUG test, a validated ambulation test, was applied to assess mobilization ability postoperatively. Data on 272 primary TKAs (including 131 with ACB and 141 with FNB) were pooled from 5 trials^16^,^17^,^20^,^23^,^33^ analyzing TUG test. While patients with ACB conducted the TUG test faster, compared with those with FNB, the finding did not reach statistical significance (MD = −35.89, 95% CI: −76.35–4.58; *P* = 0.08, Table 4).

When comparing the number of patients able to perform TUG test between groups, it was found that ACB was not significantly superior to the FNB at post-anesthesia 48 h^20^,^32^ (OR = −35.89, 95% CI: −76.35–4.58; *P* = 0.08) and 72 h^20^,^32^ (OR = −35.89, 95% CI: −76.35–4.58; *P* = 0.08). However, there were more post-TKA patients able to conduct TUG test in ACB, compared with those in FNB throughout the first 24 h (OR = 23.14, 95% CI: 5.33–100.37; *P* < 0.0001) with no heterogeneity (I^2^ = 0%, *P* = 0.91, Table 4)^16^,^32^,^33^.

##### ROM

ROM (measured with a goniometer) was utilized to evaluate mobilization ability. Data on 403 primary TKAs (including 198 with ACB and 205 with FNB) from 6 trials^17^,^18^,^24^,^25^,^32^,^33^ were analyzed in terms of ROM. The result suggested that post-TKA patients with ACB showed a better outcome in ROM comparing with those in FNB throughout the first 72 h (e.g. post-anesthesia, 24, 48, and 72 h) (Table 4).

##### The time to SLR

Data on 195 primary TKAs (including 97 with ACB and 98 with FNB) were pooled from 3 trials^18^,^19^,^33^ analyzing the time to SLR. There was no significant difference in terms of time to SLR between groups (MD = −2.91, 95% CI: −5.02–0.65; *P* = 0.13) with moderate heterogeneity (I^2^ = 75%, *P* = 0.05, Table 4).

### Opioid consumption

**Figure 6:**
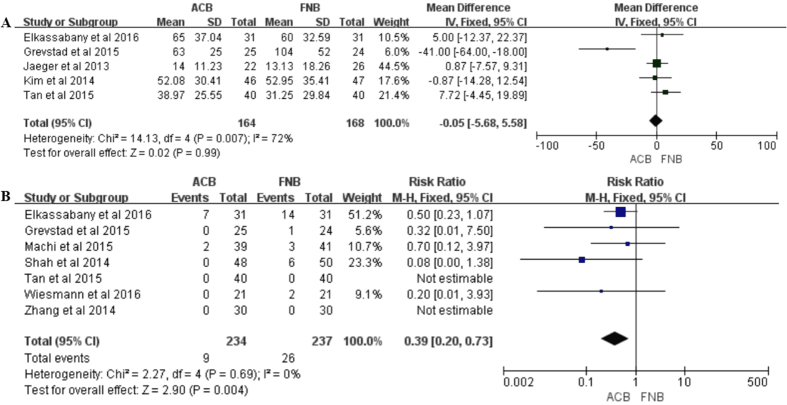
(**A**) Forest plot of opioid consumption between ACB and FNB; (**B**) Forest plot of risk of falls between ACB and FNB.

Data on 332 primary TKAs (including 164 with ACB and 168 with FNB) from 5 trials^16^,^17^,^23^,^25^,^31^ were analyzed with regard to opioid consumption. No significant difference was identified between the two groups on opioid consumption (MD = −2.93, 95% CI: −14.47–8.61; *P* = 0.62) with moderate heterogeneity (I^2^ = 72%, *P* = 0.007, Fig. 6, Table 4).

### Perioperative assessments

#### Tourniquet time

Data on 188 primary TKAs (including 94 with ACB and 94 with FNB) were pooled from 3 trials^16^,^19^,^32^ analyzing tourniquet time. There was no significant difference between the two groups on tourniquet time (MD = −1.19, 95% CI: −6.39–4.02; *P* = 0.66) with no heterogeneity (I^2^ = 0%, *P* = 0.62, Table 4).

#### Hospital stay

Data on 271 primary TKAs (including 134 with ACB and 137 with FNB) from 3 trials^25^,^31^,^33^ were analyzed with respect to the length of hospital stay. No significant difference was seen between groups regarding the hospital stay (MD = −0.86, 95% CI: −2.11–0.40; *P* = 0.18) with moderate heterogeneity (I^2^ = 82%, *P* = 0.02, Table 4).

### Patient satisfaction

Data on 194 primary TKAs (including 97 with ACB and 97 with FNB) were pooled from 3 trials^19^,^20^,^31^ analyzing patient satisfaction. The results suggested that ACB was not inferior to FNB with regard to the patient satisfaction at post-anesthesia 48 h (MD = −0.15, 95% CI: −1.29–0.98; *P* = 0.79) and 72 h (MD = 0.11, 95% CI: −1.75–1.97; *P* = 0.91). In addition, the difference between groups was not significant within post-anesthesia 24 h (MD = 0.13, 95% CI: −0.11–0.37; *P* = 0.28), and no heterogeneity was identified (I^2^ = 0%, *P* = 0.77, Table 4).

### Complication

#### Falls risk

Data on 471 primary TKAs (including 234 with ACB and 237 with FNB) from 7 trials^15^,^16^,^20^,^23^,^25^,^32^,^33^ were analyzed regarding the incidence of falls risk. There was a 70% risk reduction for ACB as compared with FNB, the finding revealed significance (OR = 0.30, 95% CI: 0.13–0.67; *P* = 0.003) with no heterogeneity (I^2^ = 0%, *P* = 0.75, Fig. 6, Table 4).

#### Other complications

When comparing adverse events between groups, there were no significant differences in the incidence of nausea and vomiting (*P* = 0.98), pruritus (*P* = 0.69), and urinary retention (*P* = 0.63), respectively. No heterogeneity was identified (Table 4).

### Sensitivity analysis

To validate our results, we further conducted sensitivity analyses to evaluate the stability of these results. Firstly, we applied leave-out method by excluding some studies to reduce between-study heterogeneity, thereby making a more robust conclusion (Tables 3 and 4). The conclusions remained unchanged in all outcomes, suggesting the stability of our meta-analysis. Secondly, due to a relatively small number of studies, REM may not be reliable, irrespective of the homogeneity test. Therefore, all outcomes initially evaluated with REM were assessed with FEM analysis, and the statistically similar results were obtained in any outcomes. Thirdly, 8 high-quality RCTs were identified, and no significant difference was identified on analysis of all outcomes for the analysis of all eligible RCTs compared to high-quality RCTs findings alone (data not shown). Therefore, our conclusion in this meta-analysis was stable and credible.

### Publication bias

**Figure 7:**
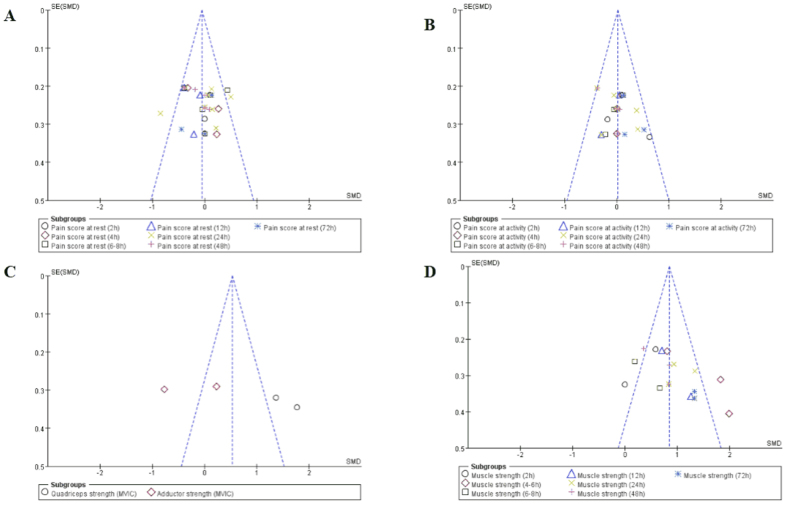
Funnel plots of primary outcomes. (**A**) For pain score at rest; (**B**) for pain score at activity; (**C**) for quadriceps and adductor strength measured by MVIC; (**D**) for quadriceps strength measured by manual 5-grade motor-strength scale.

Funnel-plot analyses on muscle strength and pain at rest or activity demonstrated symmetry, suggesting that bias was minimal (Fig. 7).

## Discussion

Total knee replacement is associated with moderate-to-severe postoperative pain, thereby effective analgesia is of extreme importance. The ideal analgesia regime that balances optimal postoperative pain control and early mobilization by preserving motor function is essential for early recovery in fast-track TKA management. FNB provides effective postoperative analgesia but reduces the strength of the quadriceps muscle and increases the risk of falling after TKA^5^,^8^,^9^. ACB after TKA is gaining popularity due to motor preservation with adequate analgesia. Several RCTs compared ACB with FNB with inconclusive outcomes^15^,^16^,^20^,^23^,^32^. Therefore, this study was performed to assess whether ACB showed superiority than FNB with respect to muscle strength, pain control, rehabilitation, and complications. The meta-analysis of RCTs provided strong evidence that ACB is an effective alternative to FNB for post-TKA patients. The ACB exhibited significantly less quadriceps strength weakness and earlier functional recovery, but provided similar analgesia and opioid consumption without increasing the incidence of complications.

Quadriceps strength for both ACB and FNB groups was reduced compared with baseline. A previous study by Jaeger *et al*. reported that the reduction of quadriceps strength from baseline was 49% with FNB but only 8% with ACB in healthy young subjects^34^. Also, a similar study on volunteers by Kwofie *et al*. demonstrated that both ACB and FNB reduced quadriceps strength balance scores compared with baseline^35^. Moreover, ACB significantly spares quadriceps motor and preserved balance compared with FNB. Therefore, peripheral nerve blockade can result in the quadriceps strength weakness. In this meta-analysis, the ACB provided significant sparing of the MVIC quadriceps strength but similar MVIC adductor strength in comparison with the FNB. In addition, quadriceps strength can be evaluated with use of five-grade motor-strength scale, and the results showed that ACB also preserved quadriceps muscle strength better than FNB at most follow-up time. One explanation is that FNB results in profound motor blockade, which has a substantial effect on quadriceps muscle strength and induces quadriceps strength weakness. However, ACB is almost a pure sensory nerve block within an aponeurotic tunnel containing several sensory nerves and only a single efferent motor nerve, which has minimal effect on quadriceps strength compared with FNB. Nevertheless, the previous meta-analysis revealed that ACB did not preserve muscle strength of adductor and quadriceps compared with FNB post TKA^21^. However, it is remarkable that two underlying factors may result in relevant deviation. Firstly, the data that were extracted from the publication by Grevstad *et al*. were wrong with respect to quadriceps strength, thereby leading to an unstable result (e.g. the standard deviation from an interquartile range was not estimated according to the Cochrane Handbook for Systematic Review of Interventions)^36^. Secondly, the pooled results with high heterogeneity (I^2^ = 87%, *P* = 0.006) found no significant association between the two groups regarding quadriceps strength, which was different from this current meta-analysis with no heterogeneity.

We adopted three indicators of mobilization ability (ROM, TUG test, and time to SLR) to assess nerve blockade following TKA. In the current study, there were significantly more post-TKA patients able to conduct TUG test within 24 h in ACB group comparing with FNB group. However, no significant difference was identified between the groups at 48 h and 72 h. Interpretation of the outcomes must be made with caution due to the relatively small sample size and different dose of the analgesia. In addition, for those patients able to perform TUG test, we were not able to show a significant difference between the two groups in mobilization ability evaluated with the TUG test. Interestingly, these findings were inconsistent with the previous meta-analyses, and we believe our meta-analysis was more accurate and comprehensive^37^. Firstly, the present study of 5 RCTs had a larger sample size than the study by Kuang *et al*.^37^, which made a more robust conclusion^37^. Secondly, sensitivity analysis was utilized to further investigate the significant heterogeneity in our study, and the results were in line with the previous analysis. Thirdly, the present study used more indicators of mobilization ability to evaluate the results. Moreover, our findings suggested that ACB showed better postoperative outcomes with respect to early functional recovery (ROM) compared to FNB post TKA, suggesting greater improvement in knee function. One possible explanation was that quadriceps strength is a factor in balance, gait, and knee function rehabilitation, and improved strength facilitates progress through physical therapy. Therefore, the relative preservation of motor strength by ACB was associated with early functional recovery in comparison to FNB.

There has been recent emphasis on whether ACB provides enough sensory coverage for TKA. Although the VAS pain scores were relatively lower in the two groups compared with baseline, no significant difference was observed in pain relief at any follow-up time between the groups, suggesting both ACB and FNB provided better analgesia without any statistical significance. It was established that small volume and low concentrations of local anesthetics substantially affected quadriceps strength and pain relief. Consequently, different doses of local anesthetics may have a minimal effect on the result. However, the optimal local anesthetic dose and concentration remain unclear and require further study. Interestingly, these findings were inconsistent with the previous meta-analyses^26^,^37^, and might be interpreted by the following reasons: Firstly, although only RCTs were included and the level of evidence was relatively high in the meta-analysis by Li *et al*., it included two studies by Kwofie *et al*. and Jaeger *et al*. comparing quadriceps strength following ACB versus FNB in volunteers without TKAs, which did not meet the inclusion criteria of the meta-analysis (RCTs comparing the analgesic effect of ACB with FNB for TKAs)^34^,^35^. Also, we strongly believe that there must be a great difference (anatomy and mechanics) between the volunteers and the patients who suffer end-stage osteoarthritis and need TKA. Secondly, the two previous meta-analyses only extracted the data regarding pain VAS scores at 0–8, 24, and 48 h post TKA, and the results showed high heterogeneity. In our meta-analysis, we evaluated the pain VAS scores at more follow-up time (e.g. post-anesthesia, 2, 4, 6–8, 12, 24, 48, and 72 h). Meanwhile, low heterogeneity was shown after regrouping. Thirdly, they did not use sensitivity analysis or subgroup to investigate the origin of high heterogeneity, thereby resulting in an unstable result. Fourthly, the study by Kuang *et al*. inappropriately combined data from RCTs and non-RCTs. Therefore, there is a need for subgroup analysis based on study design to evaluate the stability of the meta-analysis. Fifthly, the two studies did not use funnel plots to assess publication bias.

As for complications, there has been recent emphasis on risk of falls linked to peripheral nerve blocks. In our study, FNB increased the risk of falls post TKA, compared with ACB. Several studies have reported that FNB results in weakness of all four components of the quadriceps muscle increasing fall risks. On the contrary, ACB affects only vastus medialis, and thus greatly reduce the risk of falls^17^,^31^,^38^,^39^.

Opioid consumption was no different in both groups, and the incidence of postoperative nausea and vomiting was low without significant difference. This is most likely due to effective blockade reduced opioid consumption and thus minimized associated side effects of nausea and vomiting. In addition, no significant difference was identified regarding other complications with no heterogeneity, such as pruritus and urinary retention.

Our study had several strengths: Firstly, this is a comprehensive review of Level-I evidence on this topic with stricter inclusion criteria. (That is, the studies were all prospective randomized trials). Secondly, this study included four new high-quality RCTs and contained a larger sample size than the previous meta-analysis, making possible a more robust conclusion^26^. Thirdly, funnel plots analyses on primary outcomes were utilized to assess publication bias. Fourthly, sensitivity analysis through three methods was carried out to evaluate the stability of our study, and the conclusions remained unchanged in all outcomes suggesting the stability of our meta-analysis. In addition, subgroup analysis was performed to assess these results. Fifthly, we adopted the GRADE approach to assess the quality of evidence.

The limitations of this analysis include the relatively low number of patients for the ROM and patient satisfaction at different time points. In addition, another limitation is the lack of high-quality evidence in several outcomes. Furthermore, we found that heterogeneity may come from these risk factors, such as the age, gender, and dose and concentrations of local anesthetics. However, we cannot perform further analysis due to insufficient data on this topic. In addition, different anesthesia methods (spinal, general, or spinal-epidural) may influence the postoperative pain scores.

## Conclusion

Although the overall quality of the evidence can be considered “average”, we objectively assessed the benefits and risk of ACB and FNB. Based on this meta-analysis of all currently published RCTs, the findings have important implications for the medical community, namely, that ACB is an effective alternative to provide less motor strength impairment and faster recovery but provides comparable level of pain relief with decreased risk of falls in comparison with the FNB.

## Additional Information

**How to cite this article**: Wang, D. *et al*. Adductor canal block versus femoral nerve block for total knee arthroplasty: a meta-analysis of randomized controlled trials. *Sci. Rep.* **7**, 40721; doi: 10.1038/srep40721 (2017).

**Publisher's note:** Springer Nature remains neutral with regard to jurisdictional claims in published maps and institutional affiliations.

## Figures and Tables

**Table 1:** Details of meta-analyses published on this subject.

Author	Year	Studies included	Patients (knees)	Evidence	Indicators	Conclusions
Dong *et al*.	2016	6 RCTs and 2 n-RCTs	751 (751)	Level III	A, B, C, D, E, G	ACB shows no superiority than FNB group. Both of them can reduce the pain score after TKA
Li *et al*.	2015	8 RCTs^a^	434 (504)	Level II	A, B, C, D, F, G, H	ACB provide better ambulation ability, faster recovery and better pain control at rest after TKA compared to FNB
Kuang *et al*.	2015	4 RCTs and 3 CCTs	828 (828)	Level III	A, B, C, D, E, F, H	ACB results in fast pain relief and early ambulation while decreasing post-operative nausea
Li *et al*.	2015	7 RCTs and 2 n-RCTs	639 (639)	Level III	A, B, C, D, E, F	ACB preserved the strength of quadriceps more than FNB and achieves similar analgesic effects in postoperative pain

RCT, randomized controlled trial; n-RCT, non-randomized controlled trial; CCT, controlled clinical trial; ACB, adductor canal block; FNB, femoral never block; TKA, total knee arthroplasty; A, pain; B, opioid consumption; C, hospital stay; D, complications; E, muscle strength; F, risk of falls; G, MVIC of Quadriceps and Adductor; H, time up & go test.

^a^Two of these RCTs was not about TKA patients.

**Table 2:** Characteristics of the included randomized controlled trials.

Author	Year	Country	Sample size		Age (mean)		Anaesthesia	Intervention	
			ACB	FNB	ACB	FNB		ACB	FNB
Elkassabany *et al*.	2016	USA	31	31	63	65	General/Spinal	20 ml Ropivacaine 0.5% + LIF	20 ml Ropivacaine 0.5% + LIF
Wiesmann *et al*.	2016	Germany	21	21	72	66	General	Ropivacaine 0.2% + ASNB	Ropivacaine 0.2% + ASNB
Zhang *et al*.	2015	China	30	30	65	64	General	20 ml Ropivacaine 0.3%	20 ml Ropivacaine 0.3%
Tan *et al*.	2015	China	40	40	65	63	General	20 ml Ropivacaine 5 g/L, 20 ml + 0.1 mg Epinephrine + LIF	30 ml Ropivacaine 3.33 g/L + 0.1 mg Epinephrine + LIF
Memtsoudis *et al*.	2015	USA	30	29	64	64	Spinal epidural	15 ml bupivacaine 0.25%	30 ml bupivacaine 0.25%
Machi *et al*.	2015	USA	39	41	67	66	General/Spinal	Ropivacaine 0.2%	Ropivacaine 0.2%
Grevstad *et al*.	2015	Denmark	25	24	65	64	Spinal	Ropivacaine 0.2%	Ropivacaine 0.2%
Zhang *et al*.	2014	China	30	30	64	62	Spinal epidural	20 ml Ropivacaine 0.33%	20 ml Ropivacaine 0.33%
Shah *et al*.	2014	India	48	50	68	66	Spinal	20 ml Ropivacaine 0.75% + LIF	30 cc. Ropivacaine 0.75% + LIF
Liu *et al*.	2014	China	19	19	61	63	General	20 ml Ropivacaine 0.5% + LIF	30 ml Ropivacaine 0.33% + LIF
Kim *et al*.	2014	USA	46	47	68	68	Spinal epidural	15 cc Bupivacaine 0.5% + 5 μg/ml Epinephrine	30 cc Bupivacaine 0.25% + 5 μg/ml Epinephrine
Jaeger *et al*.	2013	Denmark	22	26	70	66	Spinal	30 ml Ropivacaine 0.5% + 192 ml 0.2% Ropivacaine infusion	30 ml Ropivacaine 0.5% + 192 ml 0.2% Ropivacaine infusion

ASNB, anterior sciatic nerve block; ACB, adductor canal block; FNB, femoral never block; LIF, Local infiltration analgesia.

**Table 3:** Primary outcomes of meta-analyses in included randomized controlled trials.

Variables	Studies	Patients (n)	Overall Effect		Heterogeneity P Value (I^2^)	Model
			P value	SMD/MD (95% CI)		
**Pain score at rest**						
2 h	3	167	0.76	0.05 (−0.26, 0.35)	0.95 (0%)	F
4 h	3	196	0.8	−0.04 (−0.32, 0.24)	0.14 (50%)	F
6–8 h	5	368	0.83	0.02 (−0.18, 0.23)	0.09 (50%)	F
12 h	3	216	0.07	−0.24 (−0.51, 0.02)	0.59 (0%)	F
24 h	9	612	0.9	−0.02 (−0.26, 0.23)	0.02 (58%)	R
24 h*	8	552	0.41	0.07 (−0.10, 0.24)	0.29 (18%)	F
48 h	9	612	0.25	−0.09 (−0.25, 0.06)	0.70 (0%)	F
72 h	3	160	0.76	−0.05 (−0.36, 0.26)	0.33 (11%)	F
**Pain score at activity**						
2 h	3	167	0.4	0.13 (−0.17, 0.44)	0.18 (41%)	F
4 h	2	98	1	0.00 (−0.40, 0.40)	1 (0%)	F
6–8 h	3	177	0.96	−0.01 (−0.30, 0.29)	0.67 (0%)	F
12 h	2	118	0.76	−0.06 (−0.42, 0.30)	0.38 (0%)	F
24 h	7	437	0.75	−0.03 (−0.22, 0.16)	0.24 (25%)	F
48 h	7	437	0.89	−0.01 (−0.20, 0.17)	0.42 (0%)	F
72 h	3	160	0.14	0.24 (−0.08, 0.55)	0.24 (30%)	F
**Muscle strength (5-grade scale)**						
2 h	2	118	0.24	0.34 (−0.23, 0.90)	0.14 (54%)	R
4–6 h	3	178	0.0002*	1.50 (0.70, 2.29)	0.006 (81%)	R
4–6 h*	2	98	<0.0001*	1.89 (1.40, 2.37)	0.76 (0%)	F
6–8 h	2	97	0.08	0.36 (−0.04, 0.77)	0.26 (22%)	F
12 h	2	118	<0.0001*	0.87 (0.49, 1.25)	0.20 (39%)	F
24 h	6	341	<0.0001*	0.82 (0.45, 1.18)	0.02 (62%)	R
24 h*	4	202	<0.0001*	1.09 (0.79, 1.39)	0.55 (0%)	F
48 h	6	341	<0.0001*	0.52 (0.13, 0.91)	0.008 (68%)	R
48 h*	4	241	0.003*	0.63 (0.29, 0.97)	0.28 (21%)	F
72 h	3	160	0.03*	0.91 (0.08, 1.74)	0.003 (83%)	R
72 h*	2	80	<0.0001*	1.32 (0.84, 1.81)	1.00 (0%)	F
**Quadriceps strength (MVIC)**	2	97	<0.0001*	1.55 (1.09, 2.01)	0.39 (0%)	F
**Adductor strength (MVIC)**	2	97	0.59	−0.27 (−1.25, 0.72)	0.02 (83%)	R

MVIC, maximum voluntary isometric contraction; SMD, standard mean differences; MD, mean differences; F, fixed-model effect; R, random-model effect.

^*^P values in bold denotes significance.

**Table 4:** Secondary outcomes of meta-analyses in included randomized controlled trials.

Variables	Studies (n)	Patients (n)	Overall Effect		Heterogeneity P Value (I^2^)	Model
			P value	MD/OR (95% CI)		
**Clinical outcomes**						
ROM						
24 h	4	355	<0.0001*	5.12 (2.80, 7.45)	0.35 (9%)	F
48 h	4	257	0.001*	8.39 (3.35, 13.44)	0.08 (60%)	R
48 h*	3	197	<0.0001*	11.10 (6.80, 15.40)	0.84 (0%)	F
72 h	3	197	<0.0001*	9.23 (6.45, 12.01)	0.30 (8%)	F
Patients able to perform TUG test						
<24 h	3	226	<0.0001*	23.14 (5.33, 100.37)	0.91 (0%)	F
48 h	2	121	0.58	1.19 (0.06, 24.85)	0.06 (72%)	R
72 h	2	121	0.09	0.37 (0.12, 1.17)	0.45 (0%)	F
Patients satisfaction						
<24 h	3	194	0.28	0.13 (−0.11, 0.37)	0.77 (0%)	F
48 h	3	194	0.79	−0.15 (−1.29, 0.98)	0.02 (73%)	R
72 h	2	101	0.91	0.11 (−1.75, 1.97)	0.02 (82%)	R
TUG test	5	272	0.08	−35.89 (−76.35, 4.58)	<0.0001 (98%)	R
TUG test*	4	174	0.09	−12.19 (−26.47, 2.09)	0.15 (44%)	F
The time to SLR	3	195	0.13	−2.19 (−5.02, 0.65)	0.05 (75%)	R
Tourniquet time	3	188	0.66	−1.19 (−6.39, 4.02)	0.62 (0%)	F
Hospital stay	3	271	0.18	−0.86 (−2.11, 0.40)	0.02 (82%)	R
Opioid consumption	5	332	0.62	−2.93 (−14.47, 8.61)	0.007 (72%)	R
Opioid consumption*	4	283	0.39	2.56 (−3.24, 8.37)	0.76 (0%)	F
**Complications**						
Falls risk	7	471	0.003*	0.30 (0.13, 0.67)	0.75 (0%)	F
Nausea or vomiting	4	309	0.98	1.01 (0.42, 2.45)	0.88 (0%)	F
Pruritus	3	221	0.69	1.15 (0.57, 2.32)	0.94 (0%)	F
Urinary retention	3	229	0.63	1.22 (0.54, 2.74)	0.97 (0%)	F

ROM, range of motion; SLR, straight leg raising; MD, mean differences; OR, odds ratio; F, fixed-model effect; R, random-model effect; TUG, timed-Up-and-Go.

^*^P values in bold denotes significance.
