# Author Correction: Adductor canal block versus femoral nerve block for total knee arthroplasty: a meta-analysis of randomized controlled trials

**DOI:** 10.1038/s41598-021-94766-5

**Published:** 2021-07-21

**Authors:** Duan Wang, Yang Yang, Qi Li, Shen-Li Tang, Wei-Nan Zeng, Jin Xu, Tian-Hang Xie, Fu-Xing Pei, Liu Yang, Ling-Li Li, Zong-Ke Zhou

**Affiliations:** 1 Department of Orthopedics, West China Hospital/West China School of Medicine, Sichuan University, Chengdu, 610041 People’s Republic of China; 2 Department of Prosthodontics, West China College of Stomatology, Sichuan University, Chengdu, 610041 People’s Republic of China; 3 Department of Breast Surgery, West China Hospital/West China School of Medicine, Sichuan University, Chengdu, 610041 People’s Republic of China; 4 Center for Joint Surgery, Southwest Hospital, Third Military Medical University, Chongqing, 400038 People’s Republic of China; 5 Tianjin Hospital, Tianjin, 300041 People’s Republic of China

Correction to: *Scientific Reports* 10.1038/srep40721, published online 12 January 2017.

The original version of this Article contains an error in the Abstract.

“Moreover, ACB had significantly higher risk of falling versus FNB.”

should read:

“Moreover, ACB had significantly lower risk of falling versus FNB.”

